# Unified Generative Adversarial Networks for Multidomain Fingerprint Presentation Attack Detection

**DOI:** 10.3390/e23081089

**Published:** 2021-08-21

**Authors:** Soha B. Sandouka, Yakoub Bazi, Haikel Alhichri, Naif Alajlan

**Affiliations:** Computer Engineering Department, College of Computer and Information Sciences, King Saud University, Riyadh 11543, Saudi Arabia; 439204384@student.ksu.edu.sa (S.B.S.); hhichri@ksu.edu.sa (H.A.); najlan@ksu.edu.sa (N.A.)

**Keywords:** fingerprint, liveness detection, unified generative adversarial network (UGAN), multitarget domain, compound scaling network

## Abstract

With the rapid growth of fingerprint-based biometric systems, it is essential to ensure the security and reliability of the deployed algorithms. Indeed, the security vulnerability of these systems has been widely recognized. Thus, it is critical to enhance the generalization ability of fingerprint presentation attack detection (PAD) cross-sensor and cross-material settings. In this work, we propose a novel solution for addressing the case of a single source domain (sensor) with large labeled real/fake fingerprint images and multiple target domains (sensors) with only few real images obtained from different sensors. Our aim is to build a model that leverages the limited sample issues in all target domains by transferring knowledge from the source domain. To this end, we train a unified generative adversarial network (UGAN) for multidomain conversion to learn several mappings between all domains. This allows us to generate additional synthetic images for the target domains from the source domain to reduce the distribution shift between fingerprint representations. Then, we train a scale compound network (EfficientNetV2) coupled with multiple head classifiers (one classifier for each domain) using the source domain and the translated images. The outputs of these classifiers are then aggregated using an additional fusion layer with learnable weights. In the experiments, we validate the proposed methodology on the public LivDet2015 dataset. The experimental results show that the proposed method improves the average classification accuracy over twelve classification scenarios from 67.80 to 80.44% after adaptation.

## 1. Introduction

Biometric devices use natural and inherent human characteristics or traits in identifying and verifying an individual. Biometric characteristics must be unique, universal, and permanent to each individual to constitute adequate identification, such as face, iris, retina, fingerprint, hand geometry, voice, and keystroke. The fingerprint-based biometric system was the first biometric system used for verification and identification, and it has been used for more than a century. The proven high accuracy and collectability, uniqueness, and persistence of fingerprints make the fingerprint-based biometric systems no longer limited to the security systems in government applications [1]. They are now widely used in various day-to-day personal applications and sensitive tasks such as unlocking smartphones, financial transactions, and mobile payments. The growing popularity of fingerprint-based biometric systems has led to numerous vulnerabilities, including spoofing, forgery, and presentation attacks (PAs) [2].

Presentation attacks (PAs) or spoofing attacks can be defined as “the presentation of a fake sample, such as an artifact, to the capture device with the goal of interfering with the operation of the biometric system” [3]. The threat of presentation attacks was recognized when Zwiesele et al. [4] in 2000 reported that fingerprint-stamps made of Indian rubber could fool commercial fingerprint capture devices. Then, in 2002, Matsumoto et al. [5] demonstrated that an artifact fabricated of gelatin could successfully fool most of the sensors available at the time. From then on, the researchers have initiated many efforts towards detecting and preventing presentation attacks and maintaining the security of biometric systems. 

**Figure 1:**
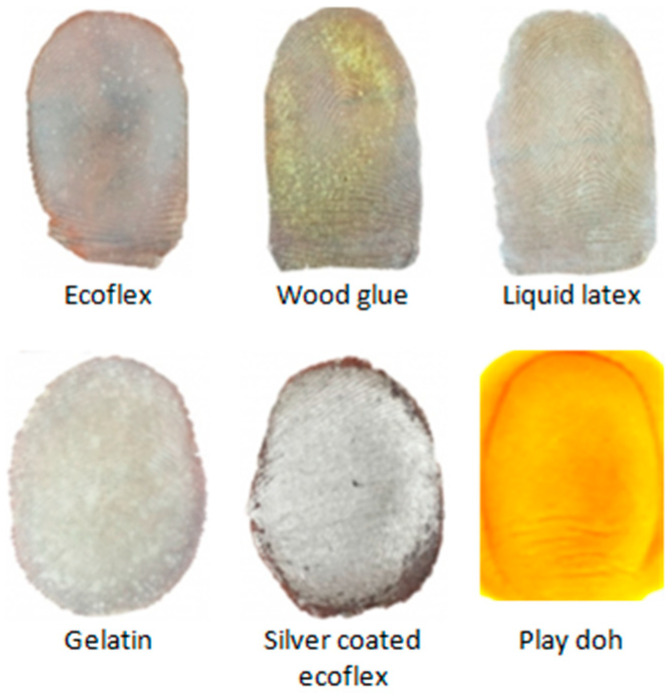
Examples of spoof artifacts fabricated using various commonly available materials [7].

The simplicity of generating an artifact has made these systems a prime target to attack. An artifact or spoof fingerprints involves the use of lifeless samples, that is, a finger from a cadaver or the use of gummy fingers, 2D or 3D printed fingerprints [6]. The spoof fingerprints can be fabricated from various commonly available materials such as Ecoflex, wood glue, latex, gelatin, silicone, and Playdoh, as shown in Figure 1.

Presentation attack detection (PAD) is an automated process for detecting and preventing PAs in a biometric system. It aims to determine if a biometric sample is bona fide (i.e., real or live) or if it is fake (i.e., artifact). These methods, also known as liveness detection methods, aim to classify a presentation as real or fake. There are numerous fingerprint PAD methods in the literature, and they can be hardware-based or software-based, depending on the technologies used to develop them. The hardware-based approaches involve adding an additional sensor to capture additional information besides the fingerprint image for the matching algorithm. A variety of physical features can be collected with additional sensors such as temperature, blood pressure, or heartbeat [8,9,10]. In general, hardware-based methods can be more accurate than software-based ones, but they are costly and complicated. Moreover, it is difficult to modify and update the hardware devices when the attacker fabricates a new type of fake fingerprint. Thus, software-based approaches have gained increasing attention.

Software-based approaches rely on image processing algorithms to analyze and extract features from the images captured by fingerprint sensors [11]. Existing software-based approaches can generally be based on handcrafted features or deep learning techniques. The earliest software-based methods proposed for liveness detection are based on handcrafted features (e.g., anatomical, physiological, or texture-based features [2]). However, the related feature-extractor methods include local binary patterns (LBPs), local phase quantization (LPQ) [12], binarized statistical image features (BSIF), and Weber local binary descriptor (WLBD). Recent solutions based on convolutional neural networks (CNNs) have been proposed to improve the detection accuracy. Compared to handcrafted-based methods, CNNs aim to learn efficient representations in an end-to-end manner.

Recently, many deep learning-based presentation attack approaches have been proposed in the literature [11,13,14,15,16,17] and have boosted the classification accuracy. However, they suffer from poor generalization ability over unknown sensors and novel materials. More specifically, we obtain high classification accuracy when a PAD network is trained and tested using images from the same sensor. However, when the network is trained on images from one sensor and tested on images from another, cross-sensor setting, we obtain low classification accuracy. 

Many researchers tried to solve accuracy degradation in fingerprint PAD in the case of novel materials and unknown sensors. Rattani et al. [18] addressed the PAD in the cross-material setting as an open-set classification problem. They proposed using a Weibull-calibrated SVM to detect PAs made of novel materials. The proposed method was conducted on the LivDet 2011 dataset and improved the detection performance by up to 44%. Ding et al. [19] proposed using a one-class support vector machine (OC-SVM) using live fingerprint samples only to generate a hypersphere boundary that comprises most of the live samples to reject spoof samples.

Nogueira et al. [20], winner of the fingerprint liveness detection competition LivDet 2015, used the transfer learning method to fine-tune VGG-19 and Alexnet on fingerprint images. The VGG model gives state-of-the-art results on the LivDet 2015 dataset with an overall accuracy of 95.51%. The proposed method failed to generalize well in cross-material and cross-sensor testing scenarios, and it was found that most generalization errors come from unknown sensors and not from novel materials. In two other works, Chugh et al. proposed training a CNN-based model on local patches of fingerprint images centered around fingerprint minutiae points. In [2], they used Inception-v3 model, and in [21], they used MobileNet-v1 for fingerprint liveness detection. The proposed methods showed a significant reduction in the error rate in cross-material and cross-sensor settings compared to the state-of-the-art PAD methods. Zhang et al. [22] developed an efficient network named light dense CNN FLDnet to improve the generalization ability of fingerprint PAD at low computational complexity. The proposed method used an attention pooling layer that overcomes the weakness of global average pooling (GAP) in most existing PAD methods. Finally, González-Soler et al. [23] proposed a new fingerprint PAD by fusing three different methods of feature encoding of dense features, namely bag-of-words (BoW), Fisher vector (FV), and vector of locally aggregated descriptors (Vlad). Then, they used a support vector machine (SVM) to classify the encoded features. The proposed approach won first place in the fingerprint liveness detection competition 2019 [24] with an overall accuracy of 96.17%.

Generative adversarial networks (GANs) are a powerful class of generative models in machine learning. They were first proposed and developed by Goodfellow et al. [25]. GANs have grown rapidly, as they are applied to different domains such as computer vision, remote sensing, natural language processing, and semantic segmentation [26]. The original GANs basically consist of two competing neural network models, namely generator and discriminator. The generator and discriminator have an adversarial relationship where they both keep competing through a two-player minimax game. The generator is trained to fool the discriminator from differentiating between real images and generated fake images. In contrast, the discriminator is optimized in order to distinguish the fake data from the real ones. 

The image-to-image translation aims to translate an image in the source domain to a corresponding image in the target domain. Most of the existing image-to-image translation approaches, whether aligned image pairs (paired) such as Pix2Pix [27] or two sets of (unaligned) sets such as CycleGAN [28], have shown promising results in image-to-image translation for two domains. Recently, StarGAN has been introduced for image-to-image translations for multiple domains with the help of only a single (unified) model.

Many researchers have used GANs to synthesize additional fingerprint images corresponding to unknown sensors and materials. Kim et al. [15] used different GAN architectures to generate artificial fingerprints along with a convolutional layer network followed by the fire module of SqueezNet for presentation attack detection. Gajawada et al. [29] proposed using a universal material translator (UMT) to generate synthetic new spoof images to train the PA classifier on and improve the performance in the cross-material setting. In another work, Chugh and Jain [30] presented a universal material generator, a style-transfer-based method, to improve generalization ability. Their proposed method improved the generalization performance of Slim-ResCNN [11], winner of fingerprint liveness detection competition 2017, in the cross-sensor scenario where the detection accuracy improved from 64.62 to 77.59%.

Recently, in [31], authors tried to address the poor generalization ability of fingerprint PAD over multiple sensors and materials by proposing a method based on transformers and CycleGAN to reduce the distribution shift between fingerprint representations coming from multiple target sensors. The proposed method improved the classification accuracy by 10.38% when trained on images from the CrossMatch sensor and the generated dataset from CycleGAN and tested using Digital Persona sensor. However, this method requires training each time on CycleGAN to convert images from one domain to another domain, which is computationally demanding and does not allow sharing information across all domains. 

In this work, we propose an alternative approach to improve the performance of fingerprint PAD over multiple sensors. One of the main advantages of this approach is its capability in generating mappings across sensors by training a single UGAN for multidomain conversion. This allows us to generate additional synthetic images for the target domains from the source domain to reduce the distribution shift between different fingerprint representations. After this step, we train an EfficientNetV2 [32] network coupled with multiple head classifiers using the source domain and the translated images. Finally, the outputs of these classifiers are aggregated using an additional fusion layer with learnable weights. In the experiments, we validate our proposed solution on the public LivDet2015 dataset. 

This work has the following contributions:

- We propose a novel domain adaptation approach for increasing the generalization ability of multiple target sensors with limited training samples using a source sensor with large labeled images.
- The method uses a UGAN model that learns across all domains using a joint optimization problem.
- Additionally, it uses a weighted fusion layer for fusing the outputs of these multiple domains.
- The experimental results show that this method can increase the accuracy up to 80.44% compared to 67.80% for the nonadaptation case.

The remaining of the paper is organized as follows: The proposed method is described in Section 2. In Section 3, we present experimental settings, and the results are presented in Section 4. Finally, conclusions are drawn in Section 5.

## 2. Proposed Method

**Figure 2:**
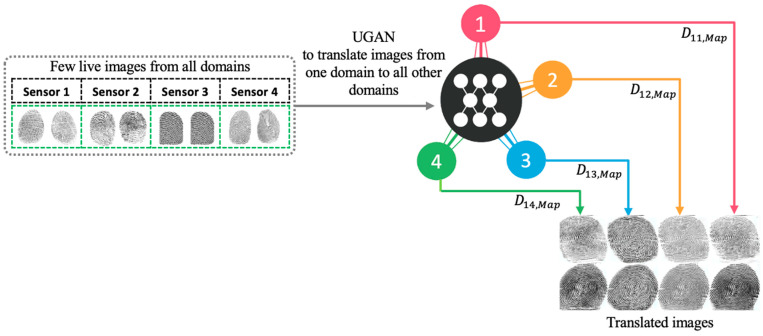
First Step: Train a translation model that learns mappings across multiple domains.

**Figure 3:**
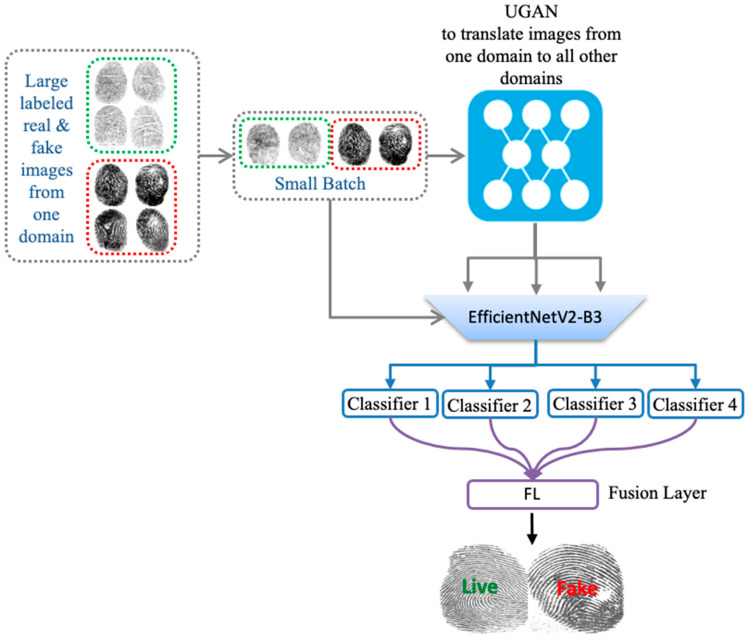
Second Step: Train CNN for feature extraction and classification.

The overall architecture of the proposed method is depicted in the figures below. First, we train a UGAN to obtain the fingerprint image-to-image translation model. This model learns the translation mapping across multiple domains (sensors) with a single generator and discriminator, as shown in Figure 2. Then, we generate additional images with sensor style translated to all other sensors. Finally, we train the pretrained CNN with both the original dataset and translated images. The CNN is followed by a set of classifiers equal to the number of sensors and a final fusion layer for the final classification, as shown in Figure 3. 

### 2.1. Multidomain Translation with UGAN

**Figure 4:**
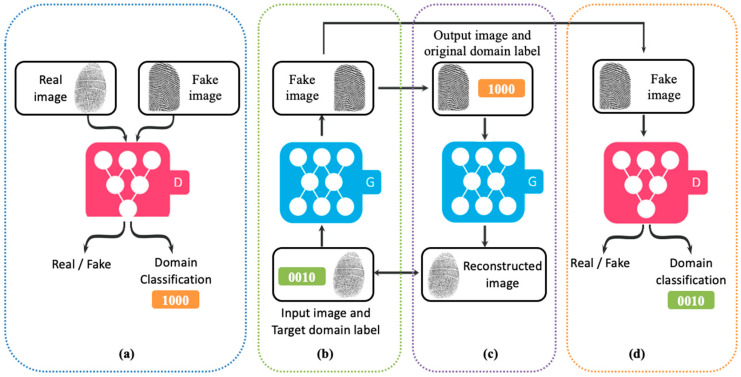
The architecture of UGAN consists of one generator G and one discriminator D. (**a**) D discriminates between real and fake images, and the real images are classified into the corresponding domain. (**b**) G takes the input image x and a randomly generated target domain label c as an input and generates a fake image. (**c**) Given the original domain label, G reconstructs the fake image into the original image (reconstruction loss). (**d**) G generates images indistinguishable from real images and classifiable as target domain by D.

The same fingerprint has different appearances in different capturing sensors. In this work, we aim to reduce the distribution shift between images from different sensors and translate an input image from one sensor to all other sensors and generate additional images. To achieve this, a UGAN (Figure 4) is utilized as the domain transfer generative adversarial network. It utilizes a single generator learning the mapping across multiple domains and discriminator with an auxiliary classifier to discriminate between fake and real images and control multiple domains simultaneously.

Let us assume that we have a set of images from *K* domains $D_{k}={\left\{X_{i}{}^{k},y_{i}{}^{k}\right\}}_{i=1}^{N_{D_{k}}}$, k=1,2,…,K. Each domain consists of $N_{D_{k}}$ images. In a multitarget setting scenario, we assume further that one of these K domains referred to as source domain has large labeled real and fake images, while the remaining domains have only few real labeled images (fixed to 25 images in our work). Our aim is to train a UGAN model that would generalize well on all sensors by taking into consideration the limited samples issue, particularly in the target sensors. Here, $X_{i}{}^{k}$ and $y_{i}{}^{k}$ represent the input fingerprint image and its corresponding label in the kth source domain and the label indicating if a fingerprint is real or fake. To this end, the dataset becomes $D=D_{k}\cup D_{k\;Map}$, where $D_{k}$ is the original fingerprint images from K domains and $D_{k\;Map}$ is the generated fingerprint images after mapping. For example, the LivDet2015 dataset has four domains for fingerprint capture sensors, namely ‘CrossMatch’, ‘GreenBit’, ‘Biometrika’, and ‘Digital Persona’. 

The generator of the translation model related to UGAN comprises two convolutional layers for downsampling, six residual blocks, and two deconvolutional layers for upsampling. The stride size is 2 for both convolutional and deconvolutional layers. The discriminator comprises an input layer, five hidden convolutional layers, and two output layers. All generator layers use instance normalization except the last output layer, and a leaky ReLU with a negative slope of 0.01 is used for the discriminator network.

The adversarial loss was adopted to generate images indistinguishable from real images, where the generator G tries to minimize the adversarial loss and the discriminator D tries to maximize it. The adversarial loss is:

(1) $${ℒ}_{\mathrm{adv}}=E_{x}\left[\log D_{\mathrm{src}}\left(x\right)\right]+E_{x,c}\left[\log(1-D_{\mathrm{src}}\left(G\left(x,c\right)\right)\right]$$

where G(x,c) is the generated image by G to fool D. To translate an input image x into an output image y and properly classify it to the target domain c, a domain classifier was added on D. The domain classification loss of real image was used to optimize the discriminator, and another domain classification loss of fake image was used to optimize the generator. The domain classification loss of real images is:

(2) $${ℒ}_{\mathrm{cls}}^{r}=E_{x,c^{\prime }}\left[-\log D_{\mathrm{cls}}\left(c^{\prime }|x\right)\right]$$

where $D_{\mathrm{cls}}\left(\;c\;\text{′}|x\right)$ represents a probability distribution over domain labels computed by D. The domain classification loss of fake images is:

(3) $${ℒ}_{\mathrm{cls}}^{f}=E_{x,c}\left[-\log D_{\mathrm{cls}}\left(c|G(x,c\right)\right]$$

D tries to minimize ${ℒ}_{\mathrm{cls}}^{r}$ to correctly classify a real image x to its corresponding original domain $c^{\prime }$, and G tries to minimize ${ℒ}_{\mathrm{cls}}^{f}$ to generate images that can be classified to its correct target domain c. Moreover, the network has reconstruction loss to preserve the content of the input images while translating the domain-related information of the image. To achieve this, a cycle consistency loss is applied to the generator. The reconstruction loss is:

(4) $${ℒ}_{\mathrm{rec}}=E_{x,c,c^{\prime }}\left[x-G{\left(G\left(x,c\right),c^{\prime }\right)}_{1}\right]$$

The full objective of the translation model is:

(5) $${ℒ}_{D}=-{ℒ}_{\mathrm{adv}}+{\;\lambda }_{\mathrm{cls}}\;{ℒ}_{\mathrm{cls}}^{r}$$

(6) $${ℒ}_{G}={ℒ}_{\mathrm{adv}}+{\;\lambda }_{\mathrm{cls}}\;{ℒ}_{\mathrm{cls}}^{f}+\lambda_{\mathrm{rec}}{ℒ}_{\mathrm{rec}}$$

where $\lambda_{\mathrm{cls}}$ and $\lambda_{\mathrm{rec}}$ control the relative importance of the domain classification and reconstruction losses, respectively, compared to the adversarial loss.

### 2.2. Multiple Classifier Fusion 

**Figure 5:**
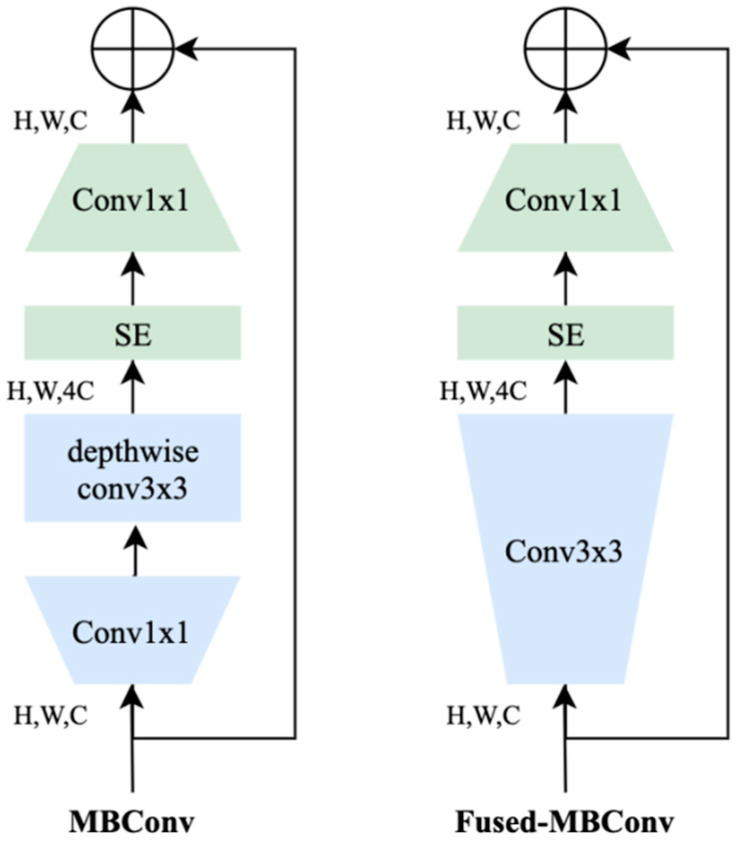
Architecture of MBConv and Fused-MBConv.

**Figure 6:**
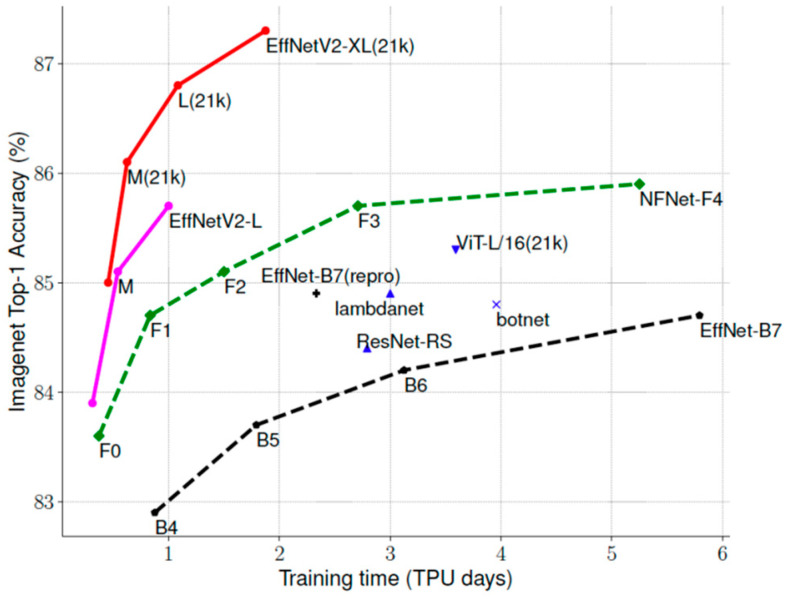
Comparison between EfficientNetV2 and other state-of-the-art CNN models in terms of ImageNet accuracy vs. training time [32].

The second component of our model is the fusion part, which contains a shared backbone (acting as a feature extractor) equipped with a weighted fusion classification layer. As a shared backbone, we use EfficientNetV2 pretrained on ImageNet dataset [32,33]. It is worth recalling that these models use Fused-MBConv that replaces the depthwise conv3×3 and expansion conv1×1 in mobile inverted bottleneck convolution (MBConv) in the original EfficientNets with a single regular conv3×3 (Figure 5). These models showed impressive results against several other CNN architectures (Figure 6). In Table 1, we report different variants with their respective parameters. In our context, we use EfficientNetV2-B3 as a good compromise between accuracy and computation complexity. 

Basically, after running the domain conversion with UGAN, we obtain a set of new images related to each domain. Then, we feed these images as input to the shared backbone for feature generation. The output of the shared EfficientNet-V2 is then fed as input to $C_{k},\;k=1,\ldots ,\;K$ classifiers with a sigmoid activation function. Each classifier $C_{k}$ gives an output probability ${\hat{y}}^{\left(t_{k}\right)}$ as follows:

(7) $${\hat{y}}^{\left(t_{k}\right)}=\sigma \left(W_{k}F_{k}(X^{\left(k\right)})\right)$$

where $W_{k}$ is the weight of each classifier $C_{k}$, σ is the sigmoid activation function, and $F_{k}(X^{\left(k\right)})$ is the feature presentations obtained from EfficientNetV2 fed with the original source images and the translated ones obtained for UGAN. Then, a weighted average fusion layer with loanable weight $w_{k}$ is used to aggregate the output of the k classifiers to generate the final probability output, i.e., live or fake.

(8) $${\hat{y}}^{\left(t\right)}=\overset{K}{\underset{k=1}{\sum }}w_{k}\;{\hat{y}}^{\left(t_{k}\right)}$$

The weights of the classifiers and the fusion layer are obtained by optimizing the well-known binary cross-entropy loss.

In Algorithm 1, we provide the main steps for training the proposed architecture for PAD.
**Algorithm 1**Input: Fingerprint image.*First Step: Train a translation model that learns transfer mappings for different domains.*-*The model was trained using the default configuration of [34] for 200 epochs using 25 real images from each domain.*-*Translate images from a source domain to that target domains to obtain a new dataset*$D=D_{k}\cup D_{k\;Map}$*.**Second Step: Train EfficientNetV2 coupled with a fusion layer for feature extraction and classification by optimizing a binary cross-entropy loss.*-*Set parameters:**Adam optimizer: learning rate: 0.0001.**Batch size = 50.*


## 3. Experiments 

### 3.1. Dataset Description 

**Figure 7:**
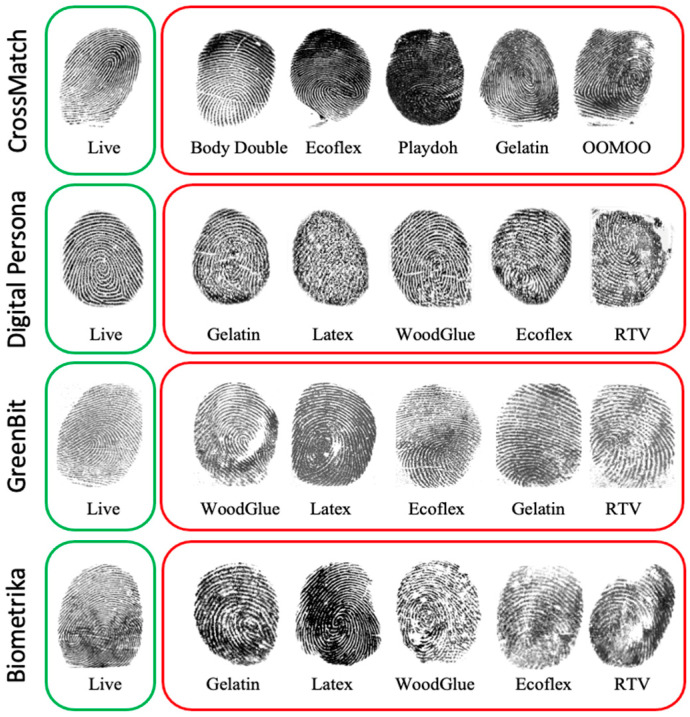
Sample images of LivDet2015 dataset. Live samples are on the left side and fake samples made of different materials are on the right side.

To evaluate and validate the proposed method, we used the public dataset provided by the Liveness Detection Competition LivDet2015 [35]. This dataset is composed of four different optical fingerprint sensors: GreenBit, Biometrika, Digital Persona, and CrossMatch. This dataset has around 19,000 images with varying sizes divided into training and testing parts. Each part has images for live and artifact fingers, as shown in Table 2. In order to mimic real scenarios, live finger images were acquired in different modes, normal mode, with wet and dry fingers, and with high and low pressure. The artifact fingerprint samples are made from a variety of materials, e.g., Playdoh, Ecoflex, gelatin, latex, and wood glue. The testing part contains images for artifact fingerprint samples made of unknown materials that do not exist in the training part, e.g., liquid Ecoflex, OOMOO, and RTV, as shown in Figure 7.

### 3.2. Experiment Setup and Performance Metrics

To evaluate the performance of the proposed methodology, we conducted different experiments on the LivDet2015 dataset. In the first experiment, we trained StarGAN to obtain a fingerprint multidomain translation model. This model generates fingerprint images with sensor style translated from one sensor to all respective sensor combinations. In the second experiment, we trained the pretrained EfficientNetV2 with both the original dataset and generated images for classification and to obtain the final fingerprint class, i.e., live or fake. All experiments were implemented in Python with the PyTorch library using a PC workstation having a Core i9 processor with a speed of 3.6 GHz, 64 GB of memory, and a GPU (with 11 GB GDDR5X memory). 

For performance evaluation, we used the standard measures proposed by Liveness Detection Competitions [35], with compatibility with ISO standard [36]:

- Accuracy: rate of correctly classified live and fake fingerprints.
- Average classification error (ACE):

(9) $$\mathrm{ACE}=\frac{\mathrm{FerrLiv}+\mathrm{FerrFake}}{2}$$

where FerrLive is the rate of misclassified live fingerprints, which is equivalent to the bona fide presentation classification error rate (BPCER). FerrFake is the rate of misclassified fake fingerprints, which is equivalent to the attack presentation classification error rate (APCER) [36]. 

## 4. Results

### 4.1. Multidomain Translation

This experiment aims to translate images from one sensor to all other sensors to generate additional images using a single translation model. We trained the translation model using randomly selected images from all domains (CrossMatch, GreenBit, Digital Persona, and Biometrika). More specifically, we randomly selected 25 real images from each domain. All images were resized to 256×256 and flipped horizontally with a probability of 0.5 for data augmentation. We trained the model using the default configuration of [34] for 200,000 iterations. In particular, we set the batch size to 16 and used the Adam optimizer with β1 = 0.5 and β2 = 0.999. We set also the parameters $\lambda_{\mathrm{cls}}=1{\;\mathrm{and}\;\lambda }_{\mathrm{rec}}=10$. Regarding the learning rate, we set it to 0.0001 for the first 100,000 iterations and set it to linearly decay for the next 100,000 iterations.

**Figure 8:**
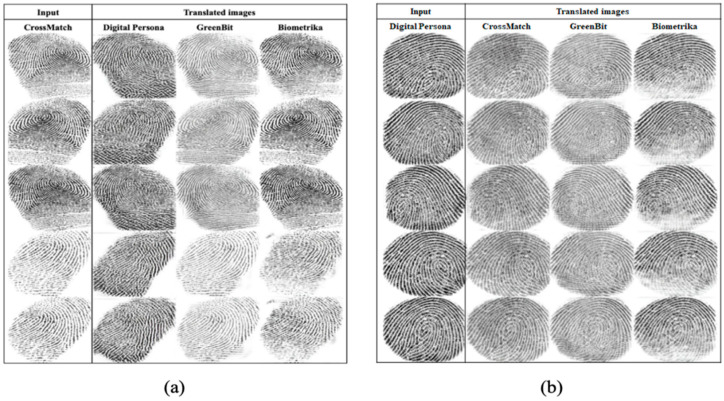
Examples of translated images from (**a**) CrossMatch and (**b**) Digital Persona to all other sensors.

**Figure 9:**
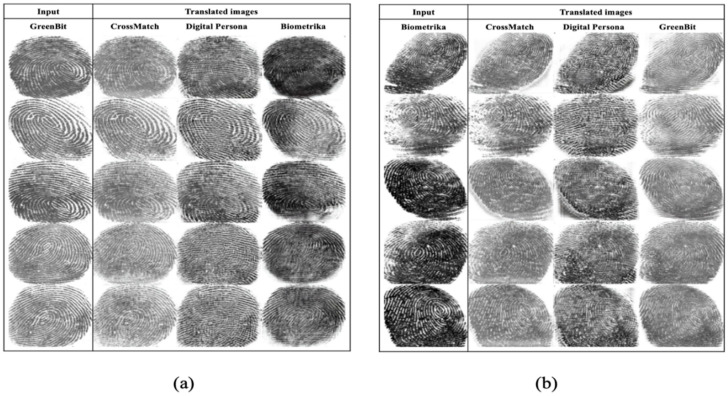
Examples of translated images from (**a**) GreenBit and (**b**) Biometrika to all other sensors.

The trained StarGAN model translates each fingerprint image from one sensor to all other sensors. For instance, if the original fingerprint image is taken from sensor 1 and the dataset contains K different sensors, then the model generates the translated images with the target sensor domain label from 2 to K. In terms of quality, all generated images mimic the target domain’s style. Figure 8 and Figure 9 show five examples of translated images from one sensor to all other sensors. The generated images are considered as new images of the LivDet2015 dataset and used in the following experiment.

**Figure 10:**
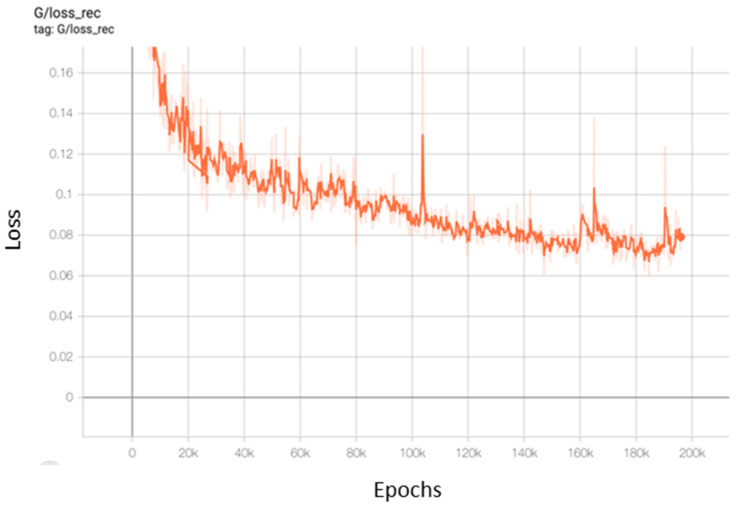
The reconstruction loss of the UGAN generator.

Figure 10 shows the evolution of the reconstruction loss of the generator of UGAN on the LivDet2015 dataset over epochs.

### 4.2. Classification

In this experiment, we employed EfficientNetV2-B3 [32] pretrained on ImageNet as the backbone network. We trained the network on images from one sensor and tested on images from another, cross-sensor setting. We used Adam optimizer as an optimization technique for training the network with the learning rate set to 0.0001 and a batch size of 50, and the number of training epochs was set to 20.

The reported results in Table 3, Table 4, Table 5 and Table 6 show that the proposed method clearly improves the PAD classification accuracy in a cross-sensor setting. For example, when we trained the proposed network on original images from GreenBit and the translated images from UGAN, the classification accuracy increased by 6.84%, 17.24%, and 13.33% for testing the network using Biometrika, Digital Persona, and CrossMatch, respectively. Moreover, the experimental results show that after adaptation the proposed method increases the average classification accuracy from 71.42 to 83.89% when using GreenBit in training, 75.09% to 82.35% when using Biometrika in training, 61.02% to 80.63% when using Digital Persona in training, and 63.71 to 74.93% when using CrossMatch in training.

Additionally, we compared our results with the proposed method in [31] which uses CycleGAN for adaptation. We can see that our method improved the average classification accuracy by 2.60%, 0.85%, 0.34%, and 0.77% when training the network using CrossMatch, Digital Persona, Biometrika, and GreenBit, respectively. In particular, from 12 scenarios, we observe that the UGAN-based method provides better results for 10 scenarios compared to the method based on CycleGAN. However, it provides lower accuracies for two cases related to Biometrika (91.20% versus 90.52%) in Table 3 and GreenBit (85.36% versus 81.12%) in Table 5. A possible explanation for this is that CycleGAN is single-domain oriented as it learns converting from one single domain to another single domain, unlike UGAN, which learns over multiple domains. On the other side, UGAN is computationally efficient compared to CycleGAN, as it takes around 48 h to translate images from one domain to all other sensors, while the latter takes about 90 h. 

## 5. Conclusions 

In this work, we have proposed a method for fingerprint presentation attack detection based on UGAN and EfficientNetV2 to improve the generalization ability. The UGAN was used to jointly learn several mappings across all domains, while EfficientNetV2 coupled with a set of classifiers and a fusion layer was used to generate the final classification result. The capability of UGAN in generating mappings across sensors using a single generator and discriminator result in reducing the computation time, where it takes around 48 h to translate images from CrossMatch to all other sensors while CycleGAN takes about 90 h. The effectiveness of the proposed method was tested by conducting multiple experiments on the LivDet2015 dataset. The experimental results prove the promising capability of the proposed method, which resulted in improving the PAD classification accuracy in a cross-sensor setting with less computation time.

## Figures and Tables

**Table 1:** Number of parameters of different EfficientNetV2 models.

Models	Parameters
EfficientNetV2-S	24 M
EfficientNetV2-M	55 M
EfficientNetV2-L	120 M
EfficientNetV2-B0	7.1 M
EfficientNetV2-B1	8.1 M
EfficientNetV2-B2	10.1 M
EfficientNetV2-B3	14.4 M

**Table 2:** Summary of LivDet2015 datasets used in the experiments.

Sensor	Image Size (px)	Resolution (dpi)	Material	Number of Training (Live/Spoof)	Number of Testing (Live/Spoof)
GreenBit	500 × 500	500	Ecoflex, gelatin, latex, wood glue, **liquid Ecoflex** *, **OOMOO** *, and RTV *	1000/1000	1000/1500
Biometrika	1000 × 1000	1000		1000/1000	1000/1500
Digital Persona	252 × 324	500		1000/1000	1000/1500
CrossMatch	640 × 480	500	Playdoh, Body Double, Ecoflex, **OOMOO** *, and Gelatin *	1500/1500	1500/1448

* The unknown materials that do not exist in the training part are in bold.

**Table 3:** The generalization performance among cross-sensors (GreenBit in training) on LivDet 2015 in terms of classification accuracy (acc%) and average classification error (ACE).

	Sensor in Testing	Biometrika		Digital Persona		CrossMatch	
Algorithm		Acc	ACE	Acc	ACE	Acc	ACE
Without Adaptation		83.68	20.20	66.60	41.75	63.97	35.47
Sandouka et al. [31]		91.20	10.20	81.20	23.21	76.96	23.06
Proposed Method		90.52	11.38	83.84	19.43	77.30	22.63

**Table 4:** The generalization performance among cross-sensors (Biometrika in training) on LivDet 2015 in terms of classification accuracy (acc%) and average classification error (ACE).

	Sensor in Testing	Biometrika		Digital Persona		CrossMatch	
Algorithm		Acc	ACE	Acc	ACE	Acc	ACE
Without Adaptation		80.12	16.76	87.28	12.33	57.86	42.81
Sandouka et al. [31]		89.52	9.81	86.72	15.30	69.77	30.62
Proposed Method		89.68	8.75	87.52	14.00	69.84	30.60

**Table 5:** The generalization performance among cross-sensors (Digital Persona in training) on LivDet 2015 in terms of classification accuracy (acc%) and average classification error (ACE).

	Sensor in Testing	Biometrika		Digital Persona		CrossMatch	
Algorithm		Acc	ACE	Acc	ACE	Acc	ACE
Without Adaptation		52.40	39.78	70.36	25.13	60.31	40.36
Sandouka et al. [31]		85.36	13.05	84.96	14.28	69.02	31.36
Proposed Method		81.12	16.15	85.36	14.21	75.40	24.79

**Table 6:** The generalization performance among cross-sensors (CrossMatch in training) on LivDet 2015 in terms of classification accuracy (acc%) and average classification error (ACE).

	Sensor in Testing	Biometrika		Digital Persona		CrossMatch	
Algorithm		Acc	ACE	Acc	ACE	Acc	ACE
Without Adaptation		70.76	26.90	70.04	29.08	50.32	44.86
Sandouka et al. [31]		80.04	17.43	76.24	22.61	60.70	35.51
Proposed Method		84.44	13.90	78.04	18.40	62.31	30.25
